# Evolutionary Origin, Gradual Accumulation and Functional Divergence of *Heat Shock Factor* Gene Family with Plant Evolution

**DOI:** 10.3389/fpls.2018.00071

**Published:** 2018-02-02

**Authors:** Xiaoming Wang, Xue Shi, Siyuan Chen, Chuang Ma, Shengbao Xu

**Affiliations:** ^1^State Key Laboratory of Crop Stress Biology for Arid Areas, College of Agronomy, Northwest A&F University, Yangling, China; ^2^State Key Laboratory of Crop Stress Biology for Arid Areas, College of Life Sciences, Northwest A&F University, Yangling, China; ^3^Center of Bioinformatics, College of Life Sciences, Northwest A&F University, Yangling, China

**Keywords:** Heat shock factors (HSFs), heat stress, abiotic stress, evolutionary origin, sequence diversification, functional divergence

## Abstract

Plants, as sessile organisms, evolved a complex and functionally diverse *heat shock factor* (HSF) gene family to cope with various environmental stresses. However, the limited evolution studies of the *HSF* gene family have hindered our understanding of environmental adaptations in plants. In this study, a comprehensive evolution analysis on the *HSF* gene family was performed in 51 representative plant species. Our results demonstrated that the HSFB group which lacks a typical AHA activation domain, was the most ancient, and is under stronger purifying selection pressure in the subsequent evolutionary processes. While, dramatic gene expansion and functional divergence occurred at evolution timescales corresponding to plant land inhabit, which contribute to the emergence and diversification of the HSFA and HSFC groups in land plants. During the plant evolution, the ancestral functions of HSFs were maintained by strong purifying pressure that acted on the DNA binding domain, while the variable oligomerization domain and motif organization of HSFs underwent functional divergence and generated novel subfamilies. At the same time, variations were further accumulated with plant evolution, and this resulted in remarkable functional diversification among higher plant lineages, including distinct HSF numbers and selection pressures of several HSF subfamilies between monocots and eudicots, highlighting the fundamental differences in different plant lineages in response to environmental stresses. Taken together, our study provides novel insights into the evolutionary origin, pattern and selection pressure of plant HSFs and delineates critical clues that aid our understanding of the adaptation processes of plants to terrestrial environments.

## Introduction

After moving to land about 400 million years ago (Ma), terrestrial plants evolved and coordinated functions at the genetic, molecular, biochemical, physiological and structural levels to adapt to the changing habitat and overcome abiotic or biotic stresses (Rensing et al., 2008; Banks et al., 2011; Jiao et al., 2011; Albert et al., 2013; Nystedt et al., 2013; Zhu, 2016). The unfavorable stresses seriously harm the growth and development of plants, with abiotic stress being the primary cause of crop loss worldwide, accounting for more than 50% yield loss of most major crop plants annually (Wang et al., 2004). To date, a large array of abiotic stress-responsive genes, along with their mechanisms of action, have been identified (Sunkar et al., 2007; Hirayama and Shinozaki, 2010; Urano et al., 2010; Kosova et al., 2011; Qin et al., 2011; Asensi-Fabado et al., 2016; Zhu, 2016), including functional and regulatory protein-encoding genes (Lata and Prasad, 2011; Shao et al., 2015). However, many of these studies focus on one particular stress condition rather than the combined effect of different stresses, which would more often be encountered in nature and leads to higher lethality in crops. Moreover, the response of plants to combined stresses differs significantly from the response to each individual type of stress (Mittler, 2006; Sewelam et al., 2014). Thus, the identification and understanding of the genes and pathways/networks involved in the response to a variety of stresses will be a critical strategy for improving crop stress tolerance.

Heat shock factors (HSFs) were initially defined as transcriptional regulators of heat shock proteins that function as molecular chaperones in protein folding and assembly and protect cells against proteotoxic damage under heat stress (HS) (Hu et al., 2009). However, accumulating evidence regarded HSFs as the core components of signal transduction chains in various abiotic stresses other than HS, suggesting that HSFs play a critical role in coordination and adaptation to multiple abiotic stresses in plants (Nover et al., 2001; Scharf et al., 2012; Guo et al., 2016). Interestingly, in non-plant organisms, the HSFs were not only required for the HS response but also participate in growth and development (Akerfelt et al., 2010; Scharf et al., 2012).

Despite considerable variability in size and sequence, plant HSFs generally contain the DNA binding domain (DBD), the oligomerization domain (OD), and flexible linker between DBD and OD regions, and are divided into several groups based on the topology of these domains. The DBD, which is characterized by a central helix-turn-helix motif and specifically binds to the HS elements in the promoters of target genes, is located close to the N-terminal of all HSFs. The OD, characterized by a bipartite heptad pattern of hydrophobic amino acid residues (HR-A/B region), is connected to the DBD by a variable length (15–80 amino acid residue) linker. Based on the length of the flexible linker between the DBD and OD regions and the number of amino acid residues inserted into the HR-A/B regions, HSFs are classified into three groups: HSFA, HSFB and HSFC. The C-terminal activation domain of the HSFA group features a short peptide motif (AHA activation domain) and is specifically presented in the HSFA group. The subfamilies of HSFB, with the exception of B5, contain a tetrapeptide (LFGV) at the C-terminus that functions as a repressor domain (RD) (Scharf et al., 2012; Guo et al., 2016). However, evolutionary studies of ancient plant HSFs are limited, and it remains to be determined which group is most ancient among HSFA, HSFB and HSFC and the subsequent evolutionary processes that occurred.

In angiosperms, HSFs have been divided into 16 subfamilies (A1–A9 in the HSFA group, B1–B5 in the HSFB group, C1–C2 in the HSFC group) (Nover et al., 2001; Hu et al., 2009; Scharf et al., 2012; Qiao et al., 2015). The biological functions of many of these HSFs in the stress response have also been identified. For example, HSFA1a in tomato plants was reported as a master regulator for acquired thermotolerance that could form a functional triad with HSFA2 and HSFB1 to affect different aspects in the HS response and recovery (Mishra et al., 2002; Liu et al., 2011). However, none of the four HSFA1 proteins in *Arabidopsis* plays a comparable role as a master regulator (Nishizawa-Yokoi et al., 2011; Scharf et al., 2012). HSFA2 is similar to HSFA1 at the structural and functional level and is the most strongly induced protein in tomato, *Arabidopsis* and rice plants under long-term or repeated cycles of HS (Heerklotz et al., 2001; Schramm et al., 2006; Charng et al., 2007; Scharf et al., 2012). The analysis of knock-out mutants and overexpression of *Arabidopsis* HSFA2 showed that this subfamily participates in several stress responses including HS, high light, oxidative stress and anoxia. Similarly, for HSFA3, as well as its role in the response to HS, it has been reported to be involved in drought stress signaling (Nishizawa et al., 2006; Ogawa et al., 2007). HSFA9 was reported to control heat shock protein expression during seed development (Kotak et al., 2007). HSFA5 acts as a specific repressor of the antiapoptotic HSFA4 (Baniwal et al., 2007), and the co-overexpression of the sunflower HSFA4a and HSFA9 genes in tobacco could improve seedling tolerance to severe dehydration and oxidative stress (Personat et al., 2014). The *HSF* gene family in mammalian contains four members and exhibits unique and overlapping functions with a great diversification in expression patterns, post-translational modifications and interacting protein partners (Akerfelt et al., 2010). Taken together, these results indicate the strong diversification of HSFs in terms of composition and function. However, the evolutionary mechanism that drives this sequence and function diversification remains to be elucidated.

Here we performed a comprehensive survey of the *HSF* gene family in 51 representative plants covering the ancient plant species to higher plants from water to land, and revealed the expansion of the *HSF* gene family, the evolutionary time points relating to the origins of each of the subfamilies and conserved motifs, sequence diversification, selection pressure variations and functional divergences. Our results present critical evidence for future experimental and evolutionary studies of HSFs as well as provide important information for understanding the adaptation of plants to terrestrial habitats.

## Materials and methods

### HSF identification and the generation of phylogenetic trees

The sequences and annotations of the representative green plants used to identify HSFs were downloaded from Phytozome v11 (https://phytozome.jgi.doe.gov/pz/portal.html) and Ensembl Plants release 31 (http://plants.ensembl.org/index.html), and the detailed information retrieved can be viewed in Table S1. The sequence of *Picea abies* was retrieved from ConGenIE (http://congenie.org/). The predicted proteome of each genome was used as a query to search for the family-specific HMM profiles of HSF_DNA-bind_PF00447 downloaded from the Pfam database (http://pfam.xfam.org/), using the Hmmsearch program in HMMER 3 (http://www.ebi.ac.uk/Tools/hmmer/). The 848 plant HSF sequences extracted from 33 plant species in the Heatster database (http://www.cibiv.at/services/hsf/) were downloaded and used in BLAST searches against the proteomes of representative plant species to identify candidate HSF proteins. Then, the two results were merged and examined for the presence of DBDs in the InterPro (http://www.ebi.ac.uk/interpro/) and PROSITE (http://prosite.expasy.org/) databases. The locations of the DBDs and ODs in the identified HSF proteins were defined by merging the results from the Pfam (http://pfam.xfam.org/), the Heatster (http://www.cibiv.at/services/hsf/), the SMART (http://smart.embl-heidelberg.de/), and the MARCOIL (http://toolkit.tuebingen.mpg.de/marcoil) programs. The AHA, nuclear localization signal (NLS), nuclear export signal (NES) and RD structures were detected based on the Heatster (http://www.cibiv.at/services/hsf/) database.

To ensure that only homologous protein sequences were used for phylogenetic tree reconstruction, the N-terminal regions (from the start of the DBD to the end of the OD) of the HSF proteins were retrieved and multiple sequence alignments were performed by MUSCLE (http://www.drive5.com/muscle/downloads.htm) with default parameters. Phylogenetic analyses were conducted by using both the NJ and the ML methods. The NJ tree was constructed by using MEGA 6 (http://www.megasoftware.net/) with 1000 bootstrap resampling, the JTT model, and pairwise deletion option. The ML tree was firstly constructed by using PhyML 3.1 (http://www.atgc-montpellier.fr/phyml/versions.php) with the JTT model selected by ProtTest 3 (http://darwin.uvigo.es/our-software/), SPRs algorithms, 16 categories of gamma-distributed substitution rates and SH-like approximate likelihood ratio test (SH-aLRT) supports. Another ML tree was constructed by using RaxML (Stamatakis, 2014) with the parameters “-T 3 -m PROTGAMMAJTT -x 12345 -N 1000 -f a.”

### Molecular evolution analysis

The protein sequence alignments and the relative cDNA sequences were converted into corresponding codon alignments by using the paraAT 2.0 program (Zhang et al., 2012). The nonsynonymous (Ka) and synonymous (Ks) rates (Ka/Ks) of each codon alignment in the paired sequences were calculated by using KaKs_Calculator 2.0 (Wang et al., 2010). The codeml program contained in the PAML 4.7 package (Yang, 2007) was used to estimate asymmetrical evolution, functional divergence and positive selection in the foreground lineages compared with the background lineages. The one-ratio model (model 0) and two-ratio model (model 2) were used to detect the different selection pressures (asymmetrical evolution) that affect specific lineages. Model 0 assumes a constant ω ratio along all branches and model 2 allows a different ratio for the foreground lineages. The site-specific null discrete model 3 and the branch-site model D were used to detect the different selective pressure affecting specific clades in which functional divergence may have occurred. The site-specific null discrete model 3 allows the ω ratio to vary among sites whilst maintaining a constant ω among the branches, and the branch-site model D allows a class of sites to be under different selection pressures between the foreground branches and the rest of the tree. The positive selection that affects some sites on specific lineages was determined by comparing the branch-site model (model A), which assumes one class of sites ω >1 in the foreground lineage, with null model A with ω fixed at one. LRTs and a computed P value were used to evaluate each examined alignment.

To unveil the correlation between sequence variation and functional divergence, the HSFs from *Arabidopsis*, rice and ancient plants were selected and compared to identify the conserved motif variations in these proteins. The software Multiple Em for Motif Elicitation (MEME) v4.11.2 (http://meme-suite.org/tools/meme) was employed to identify conserved motifs with the default parameters with minor modifications. Modifications including: (1) The optimum width of a motif was set to 6–150 amino acids to cover the DBD of HSFs. (2) The maximum number of motifs was set to 35 to identify the motifs as more as possible. The phylogenetic tree and conserved motif arrangement of these selected sequences were illustrated with iTOL v3 (http://itol.embl.de/#).

## Results

### Expansion of the *HSF* gene family with plant evolution

To investigate the origin and copy number variation of *HSF* genes during plant evolution, we conducted a comprehensive search for HSF coding genes across plant lineages, including 51 representative species from the Chlorophyta, Bryophyta, Pteridophyta, Gymnospermae, basal angiosperms, eudicots and monocots (Table S1). In total, 1213 *HSF* genes were identified and found to be present in all investigated plant lineages (Table S2 and Data S1), including *Chlamydomonas reinhardtii* and *Ostreococcus lucimarinus*, which are unicellular green alga diverged from Streptophytes over a billion years ago (Merchant et al., 2007).

**Figure 1:**
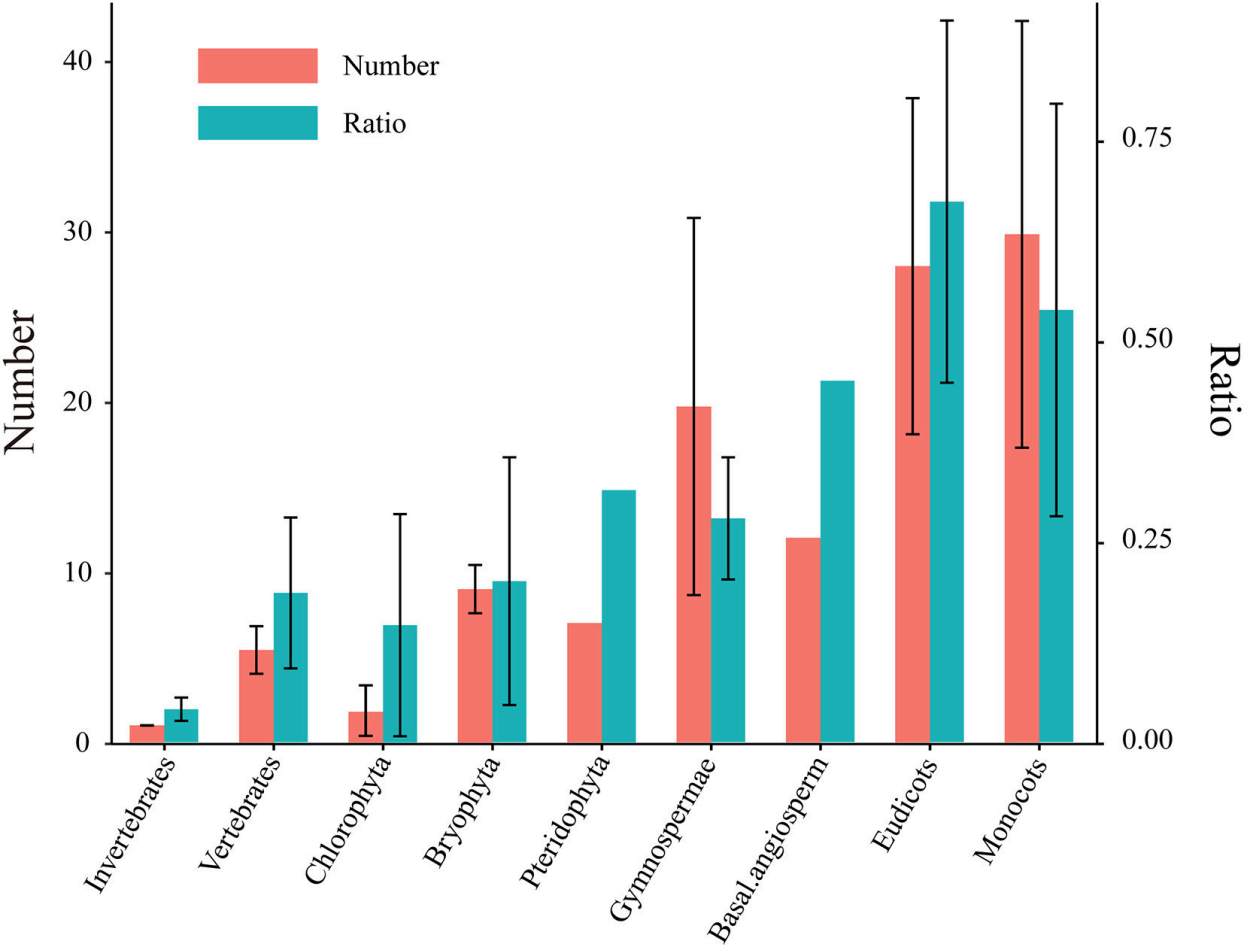
Number of HSFs and their ratios to total protein numbers in different plants. Five species in Chlorophyta, two species in Bryophyta, one species in Pteridophyta, three species in Gymnospermae, one species in basal angiosperms, 29 species in eudicots and 10 species in monocots were used to identify the *HSF* coding genes (Table S1). The left Y axis represents the identified HSF numbers. The right Y axis shows the ratio of the HSF number to the total protein number in each genome and the ratio was represented by multiplying by 1000 in this diagram. The left and right bar chart represent HSF number and ratio, respectively. All values are expressed as means ± SD.

Notably, the *HSF* gene family expanded dramatically during plant evolution, with the average number of *HSF* genes ranging from 1.8 in the Chlorophyta to 27.9 in eudicots and 29.8 in monocots (Figure 1 and Table S2). Consistent with this, the total HSF number and the ratio of HSF number to total protein number in each plant lineage revealed the significant expansion of the *HSF* gene family at three time points during plant evolution (Figure 1). At first, the average ratio of the HSF number to the total protein number increased from 0.015% in chlorophytes to 0.02% in bryophytes, which are remnants of early diverging lineages of land plants from more than 400 Ma (Rensing et al., 2008). This is consistent with the conclusions of a previous study that bryophytes increased their gene family complexity and acquired genes for tolerating environmental stresses to conquer terrestrial habitats (Rensing et al., 2008). Then, the ratio increased again in gymnosperms (from 0.02–0.028%), corresponding to the first round (ζ) of ancestral whole genome duplication (WGD) around 319 Ma (Chaw et al., 2004; Jiao et al., 2011). Finally, the massive gene expansion of the *HSF* gene family occurred in core angiosperm species, corresponding to several rounds of lineage-specific WGD (Bowers et al., 2003; Barker et al., 2009; Tang et al., 2010; Jiao et al., 2011). These outputs showed that *HSF* genes massively expanded along with WGD events and gradually accumulated in the genome during plant evolution.

### Classification and investigation into the origin of HSFs

**Figure 2:**
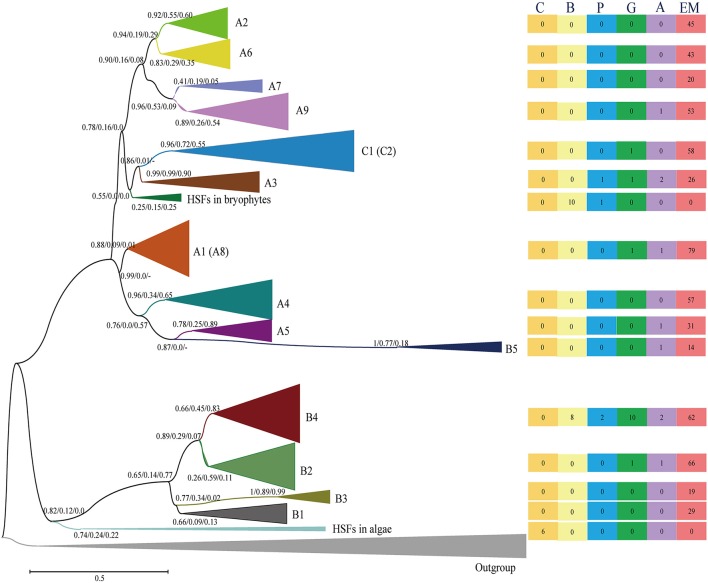
Evolutionary relationship of the HSFs from 35 plant species and four animals. The N-terminal parts of the HSFs, containing both the DBD and the OD regions, were used to construct the neighbor-joining (NJ) tree and maximum-likelihood (ML) trees with the programs of PhyML and RAxML (Figures S1–S3). The tree topology generated via the ML method with the program of “PhyML” is shown here. For the major nodes, bootstrap values of NJ tree, ML tree generated by phyML and ML tree generated by RAxML are presented from left to right. The scale bar represents 0.1 amino acid substitutions per site. The tree was arbitrarily rooted with the animal HSFs and the same type of HSFs was collapsed. The A8 subfamily was contained in A1 subfamily, and C2 subfamily was contained in C1 subfamily in phylogenetic tree. The right rectangles show the number of HSFs in different plant lineages in relative groups. The six rectangles, from left to right, present the HSF numbers in the Chlorophyta (C), Bryophyta (B), Pteridophyta (P), Gymnospermae (G), basal angiosperms (A) and angiosperms (EM), respectively. For a more detailed inspection, the fully expanded trees are provided in Figures S1, S2 and S3.

The HSF family contains several subfamilies and displays strong diversification of structure, composition and function (von Koskull-Doring et al., 2007; Scharf et al., 2012; Guo et al., 2016). To survey the evolutionary relationships of the identified HSFs, we used the neighbor-joining (NJ) and maximum-likelihood (ML) methodologies to construct phylogenetic trees for the 662 HSFs (containing the complete DBD and OD) in 35 species representing a wide variety of plant lineages (Tables S3, S4 and Data S2). The three trees (one NJ tree and two ML trees generated with different programs) showed similar topologies with minor differences, and the classifications based on the ML trees are used in this study due to their higher bootstrap values (Figure 2 and Figures S1–S3).

Generally, the HSFs of core angiosperms can be divided into known subfamilies (A1–A9, B1–B5, C1–C2), while the HSFs of more ancient plants are located at the outer branches of known subfamilies in the phylogenetic tree, providing an unprecedented opportunity to evaluate the evolutionary origin of HSF groups and subfamilies. The HSFs of chlorophytes clustered with either animal HSFs or the HSFB group (Figures S2, S3 and Table S5), suggesting that the plant HSFB group, which lacks a typical AHA activation domain, was the most ancient group among the three groups (HSFA, HSFB, and HSFC). Then, diversification was followed with the plant evolution. The HSFs in bryophytes were divided into two classes, namely BryB and BryAC, with BryB clustering with HSFB4 and BryAC clustering with the branch leading to the HSFC group and A3 subfamily. The HSFC group and A3 subfamily also lacked a typical AHA activation domain. These results suggested that the ancient plant HSFs possessed a similar sequence structure or function as animal HSFs, which are not only required for the HS response but also participate in growth and development (Scharf et al., 2012). Unexpectedly, the HSFA group, which contains major regulators in the HS response of plants, is not ancient HSFs, but rather evolved after plants moved to land.

During plant evolution, the HSFs in gymnosperms displayed further diversification (Figures S2, S3 and Table S5). In detail, some gymnosperm HSFs were clustered with the A3, B4, and B2 subfamilies, while others were clustered with the branches that lead to clades, such as C1 and C2, A4 and A5, A1, and A8, and A2, A6, A7, and A9. These diverged gymnosperm HSFs led to new origins for these HSF subfamilies, including the subfamilies that contain a typical AHA activation domain. This analysis indicates that the diversification plays an important role in the origin of HSF subfamilies in the environmental adaptation of plants to land.

### Selection pressure variations with the expansion and diversification of the HSF family

**Figure 3:**
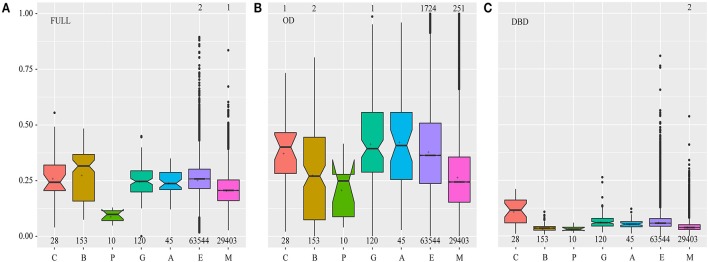
The Ka/Ks value distribution in different plant lineages. Each HSF was compared with the other HSFs in the same plant lineage one by one, and the Ka/Ks value was estimated for each compared pair. The numbers of compared pairs are illustrated at the bottom. The points which represent the Ka/Ks values of being equal to or greater than one, were not drawn and the numbers of these points were marked on the top of the relative boxplots. FULL **(A)**, OD **(B)**, and DBD **(C)** indicate the Ka/Ks values calculated based on the sequence of the full-length, OD and DBD of the HSFs, respectively. The letters C, B, P, G, A, E, and M represent the Chlorophyta, Bryophyta, Pteridophyta, Gymnospermae, basal angiosperms, eudicots, and monocots, respectively. In the boxplot, the middle line indicates the median, the box indicates the range of the 25 to 75th percentiles of the total data, the whiskers extend to data points less than 1.5 × IQR (interquartile range) away from the 1st/3rd quartile, the outer dots are outliers, the notches are defined as ±1.58*IQR/sqrt(n) and represent the 95% confidence interval for each median and the symbol “+” represents the mean value of each data set.

Natural selection shapes the function of duplicated and diversified genes, including nonfunctionalization, neofunctionalization, subfunctionalization, and functional redundancy (Flagel and Wendel, 2009; Wendel et al., 2016). To evaluate the effects of sequence diversification on functional conservation, we performed selection pressure analyses by estimating the ratio (ω) of nonsynonymous (Ka) to synonymous (Ks) substitution rates (ω = Ka/Ks) of each sequence pair in each plant lineage (Figure 3). The mean ω value was 0.24 for the full-length HSFs in all investigated species, suggesting that they were under purifying selection to maintain the important biological roles.

Significantly, natural selection distinctly varied along with plant evolution (Figure 3A). At first, the median and dispersion degree of ω values in bryophytes was larger than that in chlorophytes, highlighting that with gene duplications and sequence diversification, a considerable number of *HSF* genes experienced a shift in functional constraints to give rise to evolutionary novelties under this weaker selection pressure. Then, stronger purifying selection and more severe functional constraints acted on this family to fix their cellular functions during the later stages of plant evolution. However, many outliers were observed during the analysis of eudicots and monocots, which may have resulted from the emergence of functionally specialized subfamilies. These results suggested that HSFs may have undergone functional divergence during the adaptation of plants to land and functional specialization in angiosperms, consistent with the sequence diversification of bryophyte HSFs in the phylogenetic tree and the emergence of HSF subfamilies in angiosperms, respectively.

To further understand the evolutionary basis for the outliers in the angiosperms, we analyzed the selection pressure that acted on each HSF subfamily and the results showed that natural selection also varied among subfamilies (Figure S4). First, the subfamilies of the HSFB and HSFC groups were under more severe purifying selection than those of the HSFA group, emphasizing the sequence conservation and the earlier evolutionary origin of the HSFB and HSFC groups. Therefore, particular attention should be paid to the HSFB and HSFC groups in future HSF studies, despite the lack of a typical AHA activation domain in these two groups. Natural selection also varied among the subfamilies in the HSFA group, with the A1, A2, A5, and A6 subfamilies being subjected to more severe selection pressure than the other subfamilies. Consistent with this finding, the HSFs that could reportedly respond to HS and activate downstream genes mainly belonged to the A1, A2, and A6 subfamilies (Heerklotz et al., 2001; Mishra et al., 2002; Schramm et al., 2006; Charng et al., 2007; Liu et al., 2011; Nishizawa-Yokoi et al., 2011; Xue et al., 2014). By contrast, the A3, A4, A8, and A9 subfamilies were under weaker purifying selection allowing for the emergence of novel functionalities, as demonstrated by the fact that these subfamilies were reported to participate in several abiotic stress responses or seed development (Sakuma et al., 2006; Baniwal et al., 2007; Kotak et al., 2007; Schramm et al., 2008; Yoshida et al., 2008). For the A2, A3, A6, A7, and A8 subfamilies, significantly different selection pressures were observed between eudicots and monocots, and the A2, A3, A7, and A8 subfamilies in eudicots and the A6 subfamily in monocots were under severe purifying selection. This finding suggested that these subfamilies evolved independently in eudicots and monocots, providing insight into the fundamental basis for the observed difference in HS response between them.

To better understand the variations in selection pressure that acted on HSFs, we analyzed the selection pressures that acted on the DBD and OD of HSFs, respectively (Figures 3B,C). The DBD, which is characterized by a central helix-turn-helix motif and binds to the HS element (Guo et al., 2016), suffered severe purifying selection in all land plant lineages, implying that the HSFs maintain the ability to bind specific cis-elements and activate specific downstream genes. However, the HSFs in chlorophytes suffered weaker purifying selection than land plants, further supporting the hypothesis that HSFs experienced dramatic changes when plants moved to land. The OD, which is characterized by a leucine zipper-type protein interaction domain via which it interacts with other proteins (Scharf et al., 2012; Guo et al., 2016), was under the weaker purifying selection, which may allow HSFs to adjust to a wider range of interacting proteins and confer more flexible functions on HSFs. Notably, the variation pattern of selection pressures that acted on the OD was similar to that which acted on full-length proteins, implying that the varied OD domain takes the major selection pressure for HSF evolution during plant adaptation. These results indicate that the HSFs were constrained to bind to the specific cis-element and acquired the ability to flexibly interact with more diverse proteins during plant adaptation to terrestrial environments.

### Asymmetrical evolution, functional divergence and positive selection of HSFs during plant evolution and subfamily diversification

The *HSF* gene family is larger in plants and is involved in several regulation networks (Scharf et al., 2012; Guo et al., 2016). To further understand the functional diversification of accumulated HSFs with plant evolution, we analyzed functional divergence and positive selection by estimating the ratio (ω) of nonsynonymous (dN) to synonymous (dS) substitution rates (ω = dN/dS) under codon-based models in PAML (Yang, 2007; Table S6). In each comparison, the HSFs from more ancient plants were selected as background branches; the two-ratio branch model (model 2) indicated asymmetrical evolution, the clade model (model D) indicated functional divergence and the branch-site model (model A) detected positive selection for foreground branches.

In the above analysis, the HSFs in bryophytes experienced dramatic changes compared with the HSFs in chlorophytes. Consistent with this finding, by using the HSFs in chlorophytes as the background, asymmetrical evolution, functional divergence and positive selection were observed for the BryB and BryAC groups, suggesting that the HSFs in bryophytes experienced functional variations when plants moved to land. In addition, significant functional divergence was also detected between BryB and BryAC, suggesting that they have different biological functions. With plant evolution, no significant functional divergence or positive selection was detected in the HSFs of pteridophytes and gymnosperms, consistent with the similarity of the HSFs in pteridophytes and gymnosperms in phylogenetic tree and the selection pressure analysis.

To investigate the molecular evolutionary mechanisms for the functional diversification and specialization among HSF subfamilies in angiosperms, we also analyzed the asymmetrical evolution, functional divergence and positive selection for each subfamily, and observed many functional divergence and positive selection events (Table S6). Firstly, the B2, B3, and B4 subfamilies in angiosperms experienced significantly functional divergence events, although the HSFB group was proved to be the most ancient and conserved in the above analysis. Then, significant positive selection events were detected for the C1, A4, A5, and A8 subfamilies, consistent with their specialized functions reported in related studies (Scharf et al., 2012). Interestingly, the A3 subfamily, which was the most ancient in the HSFA group, did not experience significant functional divergence or positive selection events, indicating that the A3 subfamily in angiosperms was relatively conserved and possessed similar functions to the ancient HSFs. These results provided further clues regarding the functional roles of HSFs.

### Changes in the conserved motif composition and organization of HSFs during plant evolution

**Figure 4:**
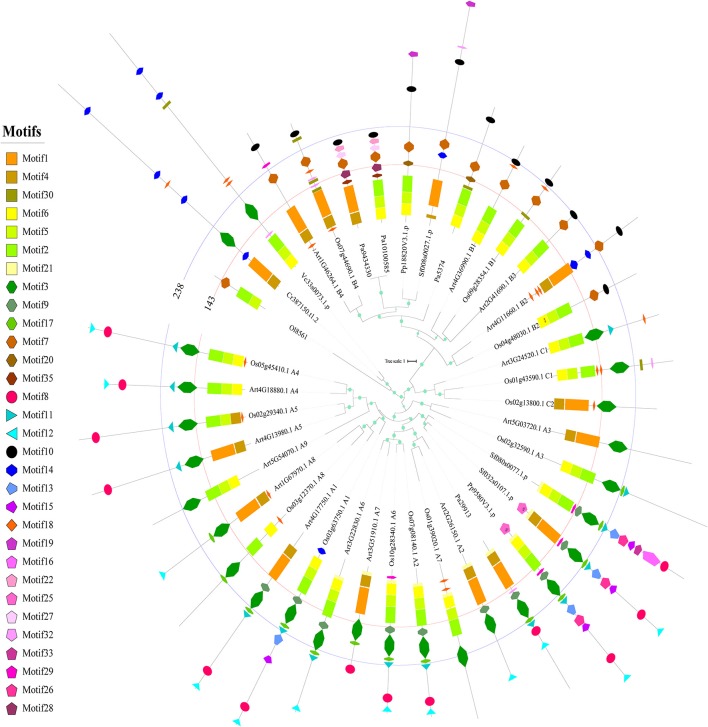
Phylogenetic relationship and conserved motifs of the selected HSFs. A total of 39 HSF proteins from *Arabidopsis*, rice and ancient plants were selected to construct the phylogenetic tree and identify the conserved motifs. The ML tree was constructed by using the full-length protein sequences. The circles on the branches indicate the SH-aLRT probabilities, and the circle size is proportional to the SH-aLRT value. The names of species are abbreviated to two or three letters and detailed information is provided in Table S3. The classifications of the selected HSFs are placed at the end of sequence names. Each conserved motif is illustrated with a specific color and shape, and the distribution of the identified motifs corresponds to their positions. The first line labels the 143rd amino acid position in all sequences, and most annotated DBDs are located in the region of 0–143 amino acids. The second line labels the 238th amino acid position in all sequences, and most annotated ODs are located in the region of 143–238 amino acids.

To better understand the basis for the sequence structure in terms of selection pressure variations and functional divergence, we further analyzed the sequence structure of the HSFs in different plant lineages and showed that the composition and organization of conserved motifs differed significantly among plants or subfamilies (Figure 4). Firstly, the HSFs in chlorophytes displayed distinct differences in motif composition, especially in the DBD and OD. One form of DBD is composed of motif 4 and motif 1 (DBD1) and the other form is composed of motif 6, motif 5, and motif 2 (DBD2). The OD also contains two different compositions, motif 3 (OD1) and motif 7 (OD2). Interestingly, the composition of the OD in BryB was based on OD2, whereas in BryAC it was based on OD1, indicating that BryB and BryAC derived from different HSF duplications of chlorophytes. Moreover, all the HSFB homologs that diverged from BryB were based on OD2, whereas all the HSFC and HSFA members that diverged from BryAC were based on OD1.

Consistent with the dramatic changes occurred in HSFs of bryophytes, the motif compositions and organizations were also greatly changed in bryophytes compared with chlorophytes. At first, motif 9 and motif 17 appeared in the OD1 domain of BryAC, and motif 8 (AHA activation domain), motif 11 (NLS), motif 12 (NES) and motifs 13, 16, and 26 (which disappeared later in evolution) firstly appeared in the sequences of BryAC. Moreover, motif 10 (the RD) and motif 19 (which disappeared later in evolution) appeared in BryB. These newly emerged motifs provide a basis for functional divergence. Notably, BryAC, as the ancestor of the HSFA and HSFC groups, contained all the motifs observed in the HSFA and HSFC members of latterly evolved plants.

In angiosperms, different HSFA subfamilies also showed differences in motif composition and organization (Figure 4). At first, the A3, A4, A5, A8, A9, C1, and C2 subfamilies, which evolved from BryAC, lacked motif 9 or motif 17 in the OD of BryAC, which was consistent with the considerable number of asymmetrical evolution, functional divergence or positive selection events and the reported functional and expressional diversification for these subfamilies^9, 31−33^. The A3, A8, and A9 subfamilies also lacked motif 8 (AHA activation domain), which was consistent with previous reports (Kotak et al., 2004; Scharf et al., 2012). Further, the motif compositions were also different between eudicot HSFs and monocot HSFs in some subfamilies, such as HSFA6 in monocots containing motif 8 (AHA activation domain), but this motif was absent from HSFA6 in eudicots. Consistent with this finding, HSFA6 in monocots was under more severe purifying selection than that in eudicots, and the expression levels of HSFA6 members in wheat were distinctly upregulated under HS (Xue et al., 2014). These results provided a detailed landscape for the variations in motif compositions and organizations during plant evolution, emphasizing the role of the OD in the evolution of the HSF family.

## Discussion

### WGDs contributed to *HSF* gene family expansion

HSFs were regarded as the terminal components of signal transduction chains that responded to various abiotic stresses and played central roles in these stress responses (Nover et al., 2001; Scharf et al., 2012; Guo et al., 2016). Our analysis indicates that HSFs were massively duplicated and gradually accumulated, and that these HSF family expansion events coincided with the WGD events that occurred during plant evolution (Jiao et al., 2011; Conant et al., 2014). We randomly selected genome fragments that contained HSF homologs and compared them by using CoGE/Gevo. A high degree of collinearity was observed (https://genomevolution.org/r/lv20 and https://genomevolution.org/r/lv1x), highlighting the roles of WGDs in HSF expansion. After ascertaining the role of WGD in HSF expansion, we needed to understand why the duplicated HSFs were not removed following diploidization, as seen for other genes, and instead accumulated in genomes. This question could be explained by a report that transcription factors, signal transducers and developmental genes were more likely to be retained in the genome after WGDs compared with other genes, resulting in the exclusive expansion of these genes (Maere et al., 2005). These ancestral WGDs and accumulated HSFs provided the foundation for sequence and function diversification and evolutionary innovation that ultimately contributed to the adaptation of plants to terrestrial habitats.

### Contrasting evolutionary histories among HSF groups

Because of the lack of an activation domain, there have been few reports on the HSFB and HSFC groups, and instead the HSFA group was regarded as being predominantly involved in the plant response to HS in previous studies. However, our analysis showed that the HSFB and HSFC groups were more ancient and under stronger purifying selection than the HSFA group, suggesting that they have important biological functions. Moreover, the motif analysis exhibited that the AHA activation domain firstly appeared in the HSFs of bryophytes, instead of being inherited from ancient HSFs. Therefore, the evolutionary origin of the AHA activation domain remains an interesting question. These results indicate that more attention should be paid to HSFB and HSFC members in future studies.

We speculate that HSFB and HSFC members could interact with HSFA members to orchestrate the functions of the latter, based on the following three reasons. First, all the subfamilies of the HSFB group, except B5, had a conserved tetrapeptide LFGV in the C-terminal domain, which is assumed to function as a repressor in the transcription network. Second, B1 in tomato plants could form a functional triad with A2 and A1, increasing their effects (Mishra et al., 2002; Liu et al., 2011). Third, the OD, which is responsible for interacting with other proteins to form heterogeneous oligomers (Scharf et al., 2012; Guo et al., 2016), underwent weaker purifying selection in the HSFA group, conferring the ability to adjust to a wider range of interacting proteins.

### Contrasting evolutionary histories of the HSF DBD and OD

It has been reported that *HSF* gene disruption in yeast is lethal even at normal growth temperatures and that the binding site targets of most *Drosophila* HSFs are actually genes encoding developmental and reproductive proteins rather than HS response genes (Wiederrecht et al., 1988; Gonsalves et al., 2011), which may represent the ancient functions of *HSF* genes. In our results, the HSFs in chlorophytes, which were closer to animal HSFs in the phylogenetic tree, suffered relatively low purifying selection in the DBD than HSFs in land plants and may maintain the ability to bind to diverse cis-elements. Significantly, the DBD in bryophytes was under stronger purifying selection than that in chlorophytes, suggesting that the range of target genes was narrowed when plants moved to land. However, the HSFs in bryophytes, which experienced sequence and motif diversification in the OD and evolved an AHA activation domain, were divided into the BryB and BryAC groups. At the same time, functional divergence and positive selection events not only occurred in the BryB and BryAC groups compared with the HSFs in chlorophytes, but also occurred between the BryB and BryAC groups. We propose that the diverse functions of ancient HSFs were assigned to diverged HSF groups when plants moved to land and that the HSFs in land plants may have evolved functional novelties, implying that the stress response system in land plants is novel.

Moreover, the selection pressure that acted on the OD was weaker than that acted on the DBD, and the selection pressure variations of the OD were consistent with the variations in full-length HSF proteins. Furthermore, the motif compositions and organizations in OD experienced dramatic changes during plant evolution. These results suggest that the functional divergence and specialization of HSFs during plant evolution mainly resulted from the evolution of the OD, which allowed HSFs to interact with a more diverse range of partners. We propose that new mutations in the OD will help plants evolve novel functions in the future in response to global warming.

### Independent evolution of HSFs in monocots and eudicots

We found that the HSFs of some subfamilies differed between monocots and eudicots. At first, lineage-specific subfamilies and varied HSF numbers were observed; For example, the A9 and B3 subfamilies were present only in eudicots, whereas the C2 subfamily was only present in monocots, and the average number of A6 subfamilies present in monocots was twice that in eudicots. Then, some families were found to be under different selection pressures; For example, the A2, A3, A7, and A8 subfamilies in eudicots and the A6 subfamily in monocots were under more severe purifying selection than in eudicots. The results indicated that these subfamilies may have different functions between monocots and eudicots, suggesting distinct HS response networks in these two plant lineages. We should therefore pay more attention to this difference in future HSF studies and design different strategies to enhance thermotolerance depending on the plant species.

## Author contributions

XW, XS, and SC carried out the public genome data collection. XS and XW performed the data analyses. XW, CM, and SX contributed to the study design. XS, XW, and SX wrote the manuscript. All authors were involved in the revision of the manuscript and approved the final manuscript.

### Conflict of interest statement

The authors declare that the research was conducted in the absence of any commercial or financial relationships that could be construed as a potential conflict of interest.
