# Supplementary material for: Evolutionary Origin, Gradual Accumulation and Functional Divergence of Heat Shock Factor Gene Family with Plant Evolution
Source: Front Plant Sci. 2018 Feb 2;9:71. doi: 10.3389/fpls.2018.00071 (PMC5801592; doi:10.3389/fpls.2018.00071)
Supplement: Supplementary file 7 [file Presentation1.PDF]

*Supplementary Material*

# Evolutionary origin, gradual accumulation and functional divergence of *heat shock factor* gene family with plant evolution

Xue Shi[1†], Xiaoming Wang[1†], Siyuan Chen[2,3], Chuang Ma[2,3], Shengbao Xu[1*]

[1]*State Key Laboratory of Crop Stress Biology for Arid Areas, College of Agronomy, Northwest A&F University, Yangling 712100, Shaanxi, China.*

[2]*State Key Laboratory of Crop Stress Biology for Arid Areas, College of Life Sciences, Northwest A&F University, Yangling 712100, Shaanxi, China.*

[3]*Center of Bioinformatics, College of Life Sciences, Northwest A&F University, Yangling 712100, Shaanxi, China.*

†These authors have contributed equally to this work.

*** Correspondence:**
Shengbao Xu
xushb@nwsuaf.edu.cn

## 1 Supplementary Tables

Table S1. The species and their genome information used for *HSF* gene identification.

Table S2. The identified *HSF* genes and their annotations based on PFAM and Heatster databases.

Table S3. The species used for phylogenetic tree construction.

Table S4. The HSFs used for phylogenetic tree construction.

Table S5. The distribution of the HSFs of ancient plants in NJ and ML tree.

Table S6. The *P* values of different codon substitution based evolution models.

## 2 Supplementary Figures

Figure S1. The detailed NJ tree of HSFs. The N-terminal parts of HSFs (from the start of the DBD to the end of the OD) were aligned and constructed as an NJ tree. The classification of HSFs was based on HSF annotations and phylogenetic relationships and is depicted on the outer layer. The names of HSFs in the different plant lineages are illustrated with different colors, and the gray, red, orange, yellow, green, brown, light purple and light blue colors represent the HSFs from animals, Chlorophyta, Bryophyta, Pteridophyta, Gymnospermae, basal angiosperms, eudicots and monocots, respectively. The names of species are abbreviated to two or three letters and detailed information is provided in Supplementary Table S3. The subfamily classifications in Heatster (http://www.cibiv.at/services/hsf/) database are placed at the end of sequence names. The bootstrap values are plotted as circles at the nodes, with the circle size proportional to the bootstrap value. The tree was arbitrarily rooted using the animal HSFs as outgroup.

Figure S2. The detailed ML tree of HSFs constructed with the program of PhyML. The N-terminal parts of HSFs (from the start of the DBD to the end of the OD) were aligned and constructed as an ML tree. The classification of HSFs was based on HSF annotations and phylogenetic relationships and is depicted on the outer layer. The names of HSFs in the different plant lineages are illustrated with different colors, and the gray, red, orange, yellow, green, brown, light purple and light blue colors represent the HSFs from animals, Chlorophyta, Bryophyta, Pteridophyta, Gymnospermae, basal angiosperms, eudicots and monocots, respectively. The names of species are abbreviated to two or three letters and detailed information is provided in Supplementary Table S3. The subfamily classifications in Heatster (http://www.cibiv.at/services/hsf/) database are placed at the end of sequence names. The circles on the branches indicate the SH-aLRT probabilities, and the circle size is proportional to the SH-aLRT value. The tree was arbitrarily rooted using the animal HSFs as outgroup.

Figure S3. The detailed ML tree of HSFs constructed with the program of RaxML. The N-terminal parts of HSFs (from the start of the DBD to the end of the OD) were aligned and constructed as an ML tree. The classification of HSFs was based on HSF annotations and phylogenetic relationships and is depicted on the outer layer. The names of HSFs in the different plant lineages are illustrated with different colors, and the gray, red, orange, yellow, green, brown, light purple and light blue colors represent the HSFs from animals, Chlorophyta, Bryophyta, Pteridophyta, Gymnospermae, basal angiosperms, eudicots and monocots, respectively. The names of species are abbreviated to two or three letters and detailed information is provided in Supplementary Table S3. The subfamily classifications in Heatster (http://www.cibiv.at/services/hsf/) database are placed at the end of sequence names. The circles on the branches indicate the SH-aLRT probabilities, and the circle size is proportional to the SH-aLRT value. The tree was arbitrarily rooted using the animal HSFs as outgroup.

Figure S4. The Ka/Ks value distribution of the different HSF subfamilies in angiosperms. Each HSF was compared with the other HSFs in the same subfamily one by one, and the Ka/Ks value was estimated for each compared pair. The numbers of compared pairs are illustrated at the bottom of each boxplot. The points which represent the Ka/Ks values of being equal to or greater than one, were not drawn and the numbers of these points were marked on the top of the relative boxplots. FULL, DBD and OD indicate the Ka/Ks values calculated based on the sequence of the full-length, OD and DBD of the HSFs, respectively. The letters E and M in the X-axis labels represent eudicots and monocots, respectively. For an explanation of each part of the boxplot, please refer to the legend of Figure 3.

## 3 Supplementary Data

Data S1. The protein sequences of identified HSFs in Fasta format. The sequence names derived from the Supplementary Table S2.

Data S2. The protein alignment file used for phylogenetic tree construction.
