# Supplementary figures and images for: Evolutionary Origin, Gradual Accumulation and Functional Divergence of Heat Shock Factor Gene Family with Plant Evolution
Source: Front Plant Sci. 2018 Feb 2;9:71. doi: 10.3389/fpls.2018.00071 (PMC5801592; doi:10.3389/fpls.2018.00071)

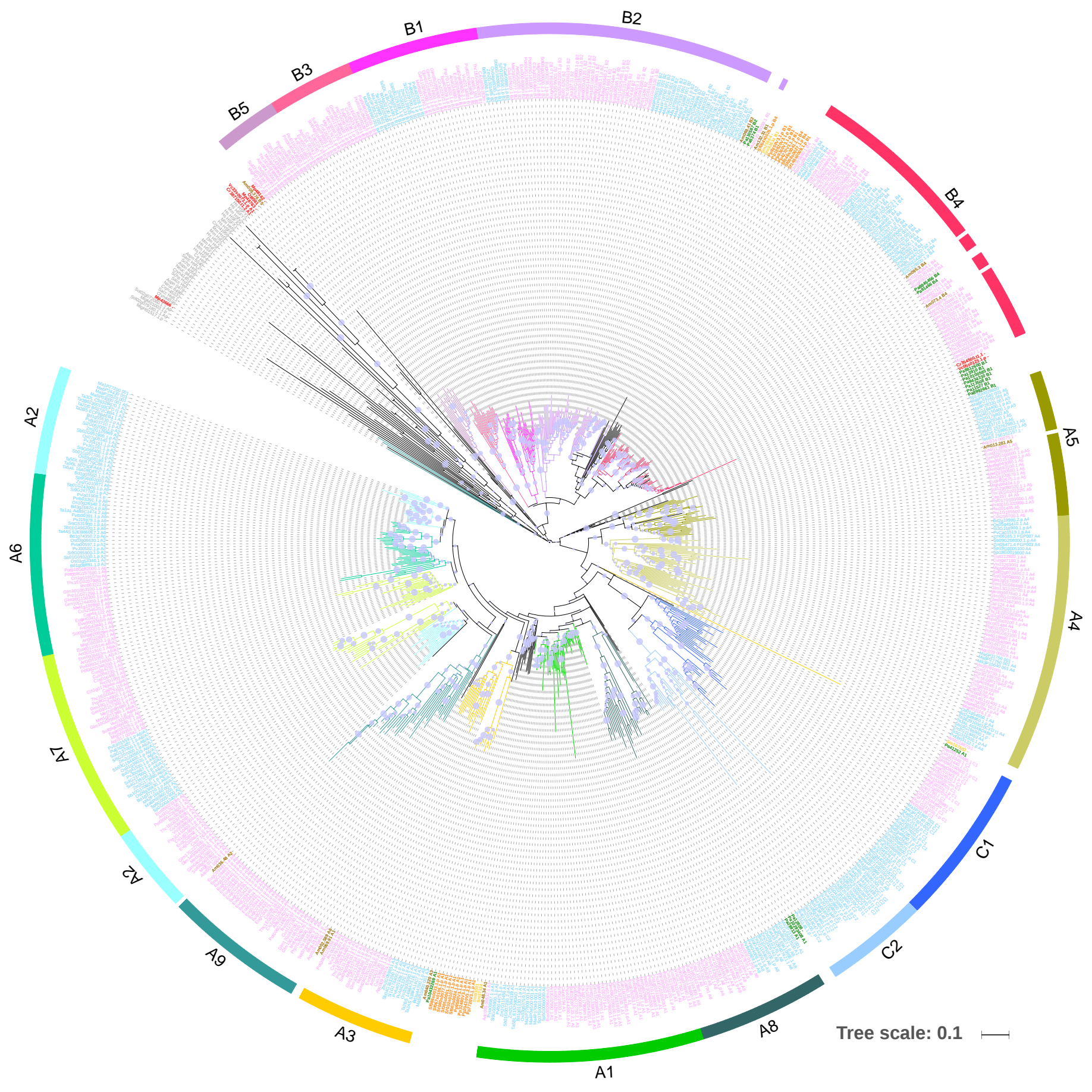

Supplement: Supplementary file 10 [file Image1.PDF]

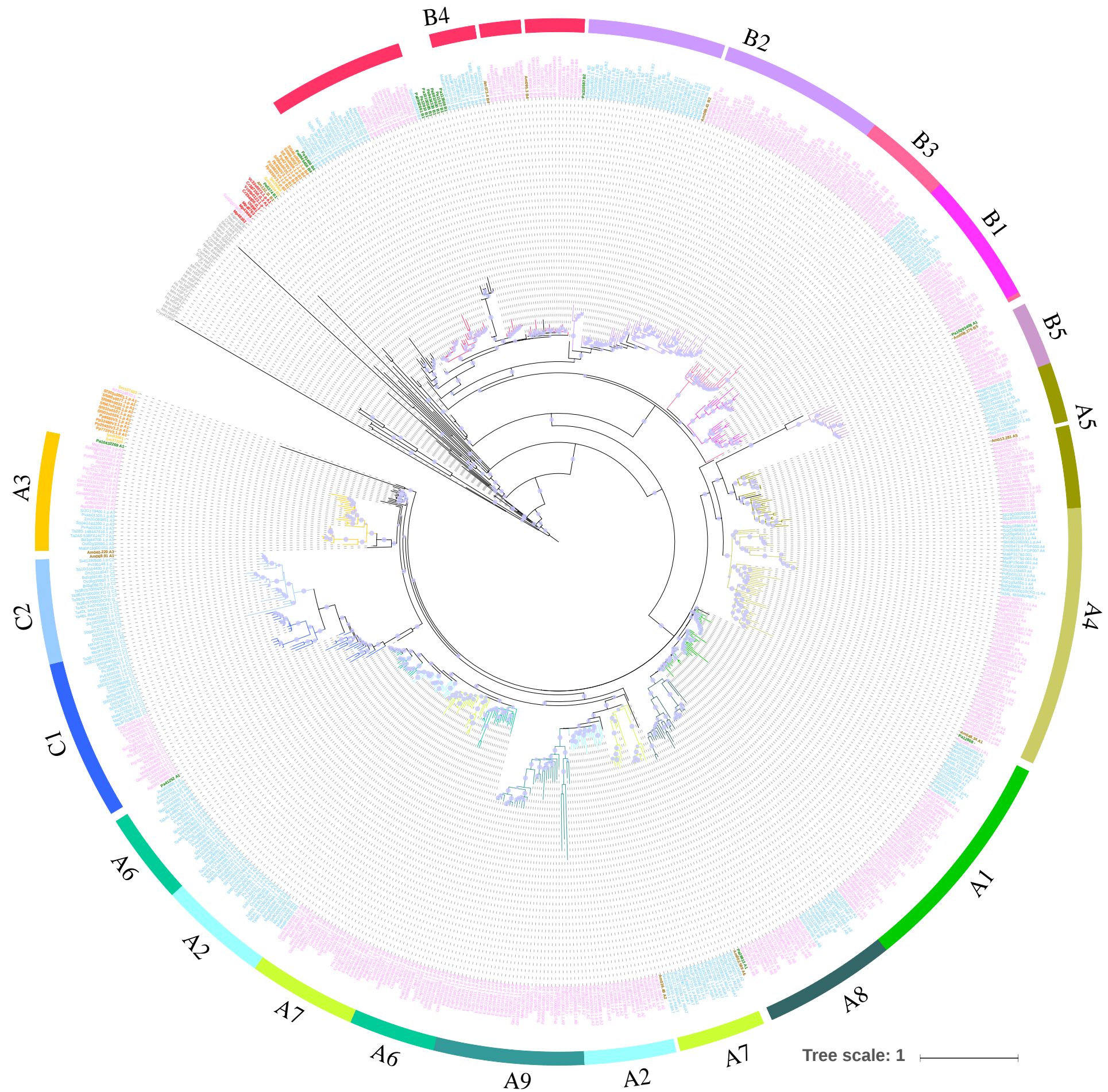

Supplement: Supplementary file 11 [file Image2.PDF]

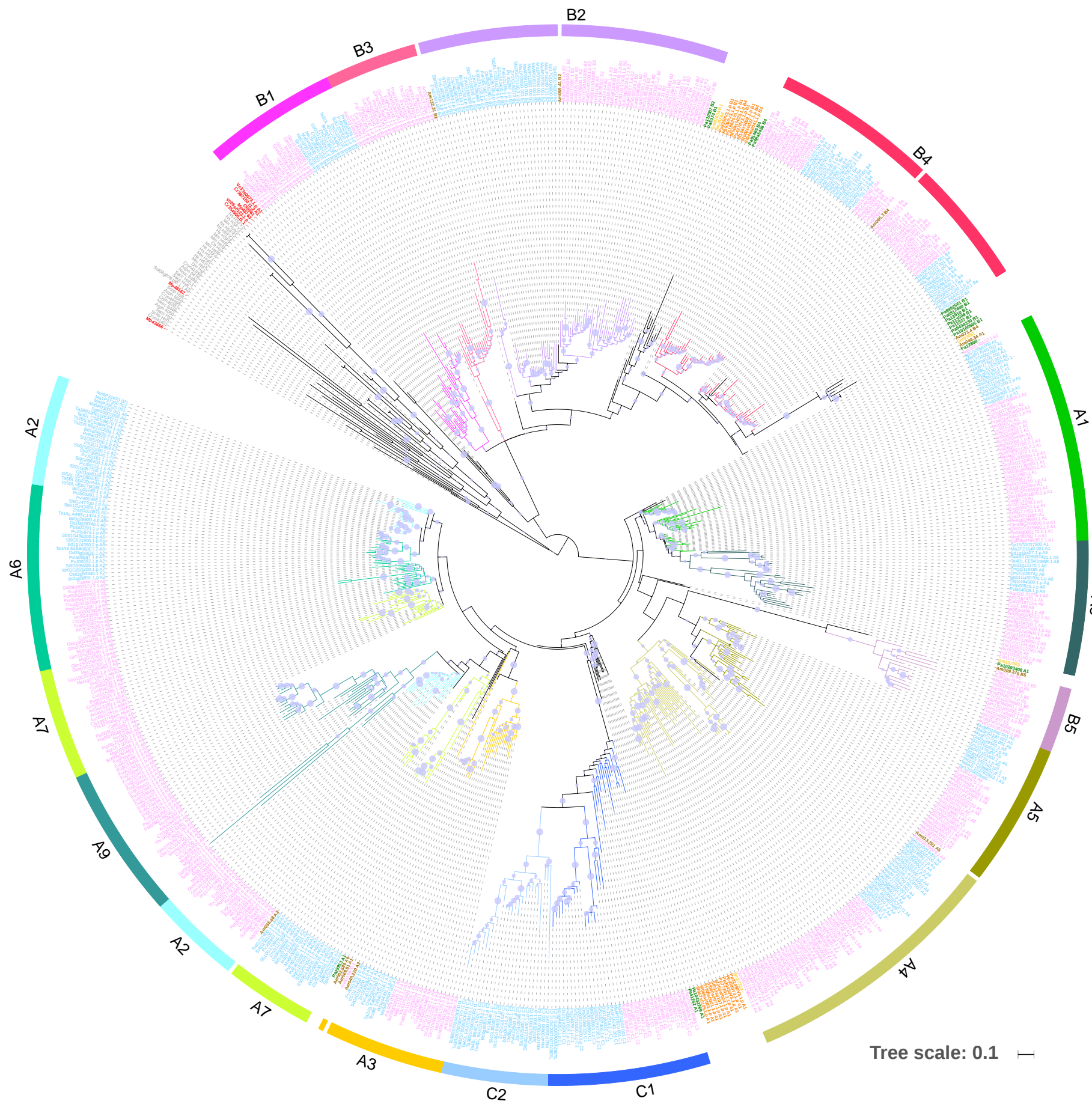

Supplement: Supplementary file 12 [file Image3.PDF]

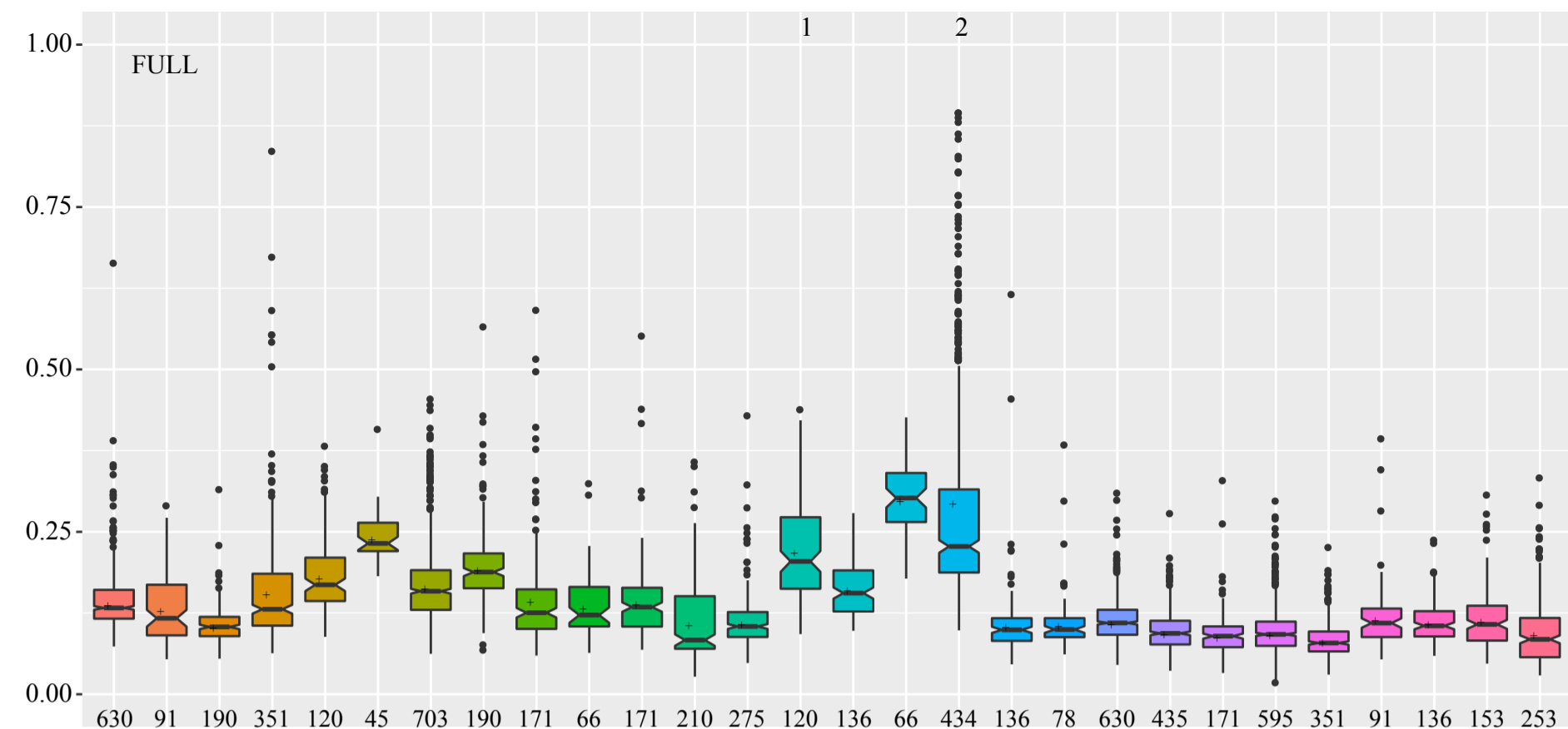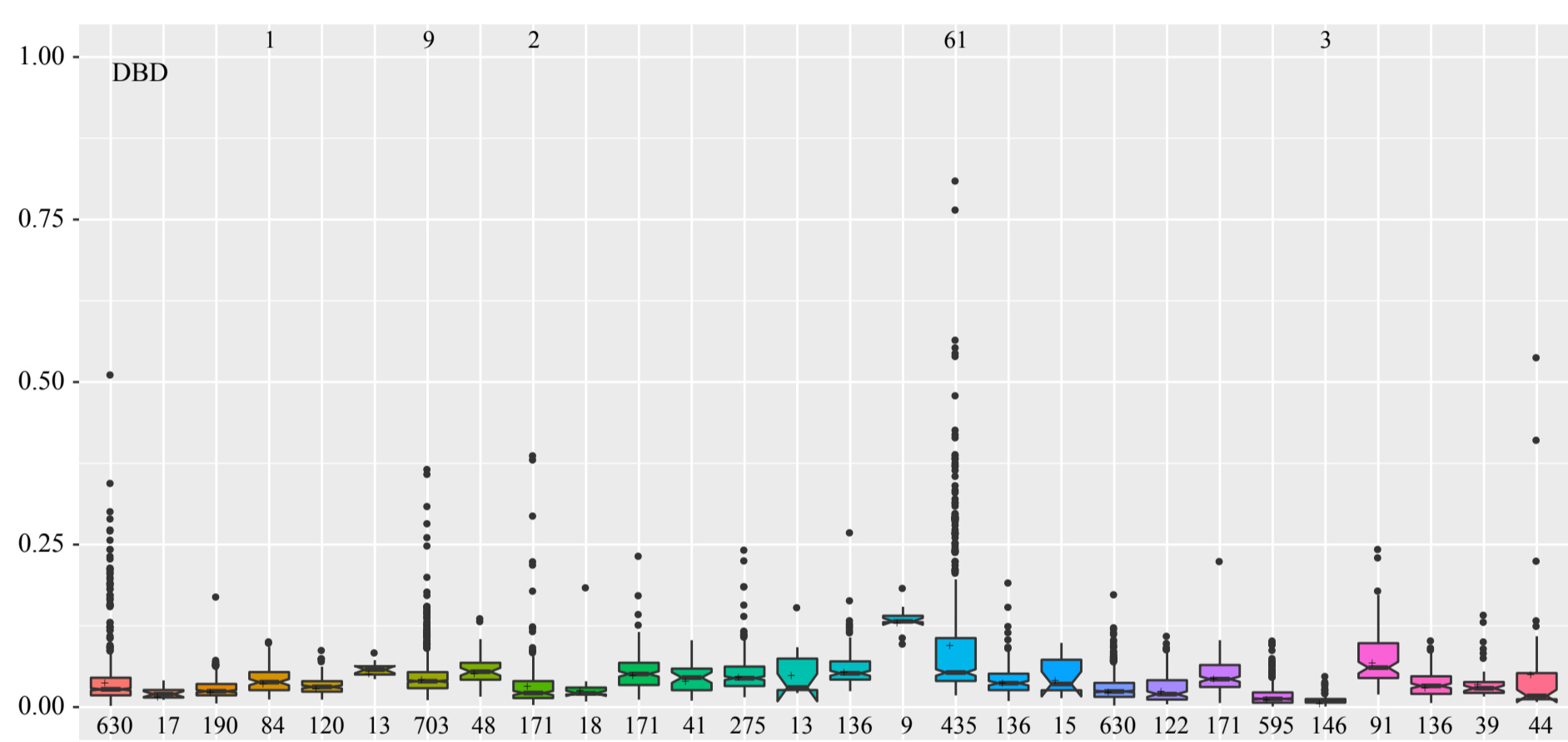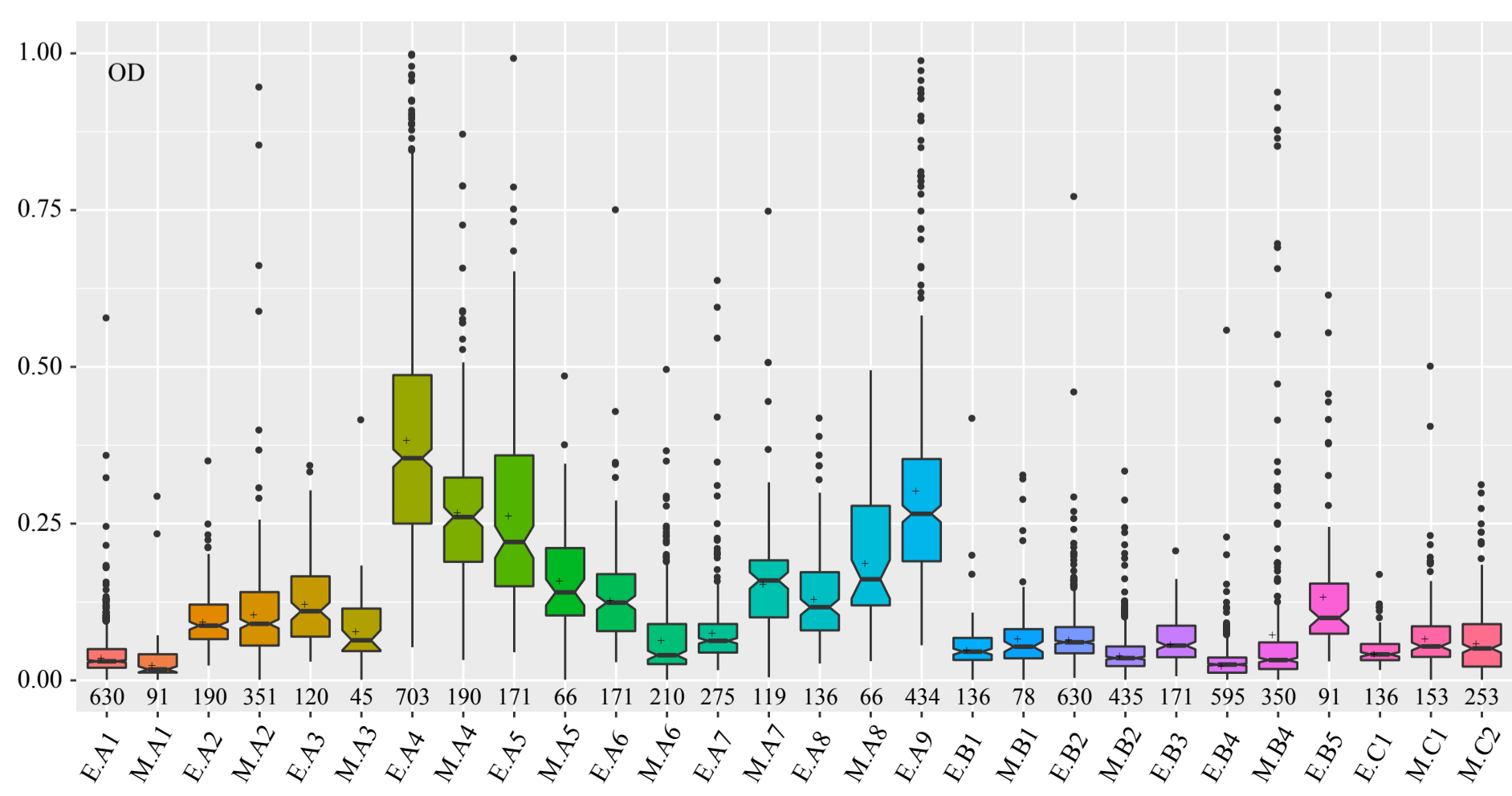

Supplement: Supplementary file 13 [file Image4.PDF]
